# Characterization of imported malaria, the largest threat to sustained malaria elimination from Sri Lanka

**DOI:** 10.1186/s12936-015-0697-0

**Published:** 2015-04-24

**Authors:** Priyani Dharmawardena, Risintha G Premaratne, WM Kumudunayana T de AW Gunasekera, Mihirini Hewawitarane, Kamini Mendis, Deepika Fernando

**Affiliations:** Anti Malaria Campaign Headquarters, Public Health Complex, Narahenpita, Colombo, 5 Sri Lanka; ᅟ, No.141 Jawatta Road, Colombo 5, Sri Lanka; Department of Parasitology, Faculty of Medicine, University of Colombo, Kynsey Road, Colombo, 8 Sri Lanka

**Keywords:** Sri Lanka, Malaria, Elimination, Imported malaria, Prevention of reintroduction, Challenges to sustain malaria elimination

## Abstract

Sri Lanka has reached zero indigenous malaria cases in November 2012, two years before its targeted deadline for elimination. Currently, the biggest threat to the elimination efforts are the risk of resurgence of malaria due to imported cases. This paper describes two clusters of imported malaria infections reported in 2013 and 2014, one among a group of Pakistani asylum-seekers resident in Sri Lanka, and the other amongst local fishermen who returned from Sierra Leone. The two clusters studied reveal the potential impact of imported malaria on the risk of reintroducing the disease, as importation is the only source of malaria in the country at present. In the event of a case occurring, detection is a major challenge both amongst individuals returning from malaria endemic countries and the local population, as malaria is fast becoming a “forgotten” disease amongst health care providers. In spite of a very good coverage of diagnostic services (microscopy and rapid diagnostic tests) throughout the country, malaria is being repeatedly overlooked by health care providers even when individuals present with fever and a recent history of travel to a malaria endemic country. Given the high receptivity to malaria in previously endemic areas of the country due to the prevalence of the vector mosquito, such cases pose a significant threat for the reintroduction of malaria to Sri Lanka. The challenges faced by the Anti Malaria Campaign and measures taken to prevent the resurgence of malaria are discussed here.

## Background

The decline in the number of malaria cases between the years 2000–2007 led to the commencement of pre-elimination malaria efforts in Sri Lanka. This together with the end of the civil conflict in May 2009 made it possible to strengthen surveillance, prevention and control efforts throughout the country, thus rapidly changing the epidemiology of malaria. During the period 2008–2012, the objectives of the national programme were the elimination of indigenous *Plasmodium falciparum* infections by end 2012 in non-conflict and transitional areas and the elimination of indigenous *Plasmodium vivax* infections by the year 2012 in 75% of non-conflict and transitional areas of the country, while sustaining zero mortality due to malaria [1]. With the end of the civil conflict, these objectives were reformulated as elimination of indigenous *P. falciparum* by the end of 2012 and indigenous *P. vivax* by the end of 2014 [2]. Both these objectives have thus far been achieved with the last reported case of indigenous malaria being reported in October 2012. Currently, the biggest threat to the elimination efforts is the risk of resurgence due to imported malaria and the continuing receptivity in several parts of the country due to the persistence of malaria vectors.

Over the past six years, most of the imported malaria cases were being reported from foreign travellers or Sri Lankan nationals returning from malaria endemic countries, mainly India [3,4]. With enhanced parasitological surveillance, the highest number of cases was reported in 2013 (95 cases: 44.2% by *P. falciparum*, 54.7% by *P. vivax*, 1.1% by other species) whereas 49 were reported in 2014. This study reports on two clusters of imported malaria, one amongst Pakistani asylum-seekers resident in a malaria non-endemic area in Sri Lanka and the other amongst local fishermen who returned from Sierra Leone, the statistics of which added to the burden of imported malaria in Sri Lanka in 2013/14. The implications of the imported cases are discussed in the context of the challenges faced by the Anti Malaria Campaign (AMC) and measures taken to prevent the reintroduction of malaria.

## Findings

### Cluster of malaria infections amongst Pakistani asylum seekers in Sri Lanka

Two children were admitted with fever to a District General Hospital (DGH) situated in a traditionally malaria non-endemic area of Sri Lanka in the Western Province and diagnosed with *P. vivax* malaria on 8^th^ and 12^th^ of July 2013, respectively. Extensive field and outbreak investigation was immediately initiated after the first case was reported, which led to a coordinated effort between the AMC Headquarters (AMC HQs) and religious leaders under whose patronage this Pakistani community was settled.

Investigations revealed that they were asylum seekers who had been already registered with the United Nations High Commission for Refugees (UNHCR). It was also noted that the group was expanding due to new arrivals. Between July and December 2013, four active case detection (ACD) programmes were carried out amongst them, the largest being the initial programme screening 839 individuals (from a total of approximately 1000 Pakistani nationals) and lasting four days. All of them were asymptomatic at the time of screening. In order to ensure maximum sensitivity, microscopy and Rapid Diagnostic Tests (RDTs) were carried out in each of these screening programmes that were conducted at 4–6 week intervals. This included the screening of all persons who were registered under the UNHCR during the first screening, and in subsequent screening all those who were reported positive or missed in the previous screening and new arrivals since the last screening, regardless of their clinical symptoms.

Five malaria positive infections, including a 31-year-old woman in her 32^nd^ week of pregnancy was diagnosed by active surveillance. None of these individuals were symptomatic at the time of diagnosis. However all of these persons gave a history of fever and visiting a government hospital or General Practitioner approximately 1–2 weeks prior to diagnosis of disease. On further inquiry it was revealed that despite the fact that these patients had arrived from a malaria endemic country, none of the health care providers had requested a blood smear for malaria parasites to be carried out.

**Figure 1:**
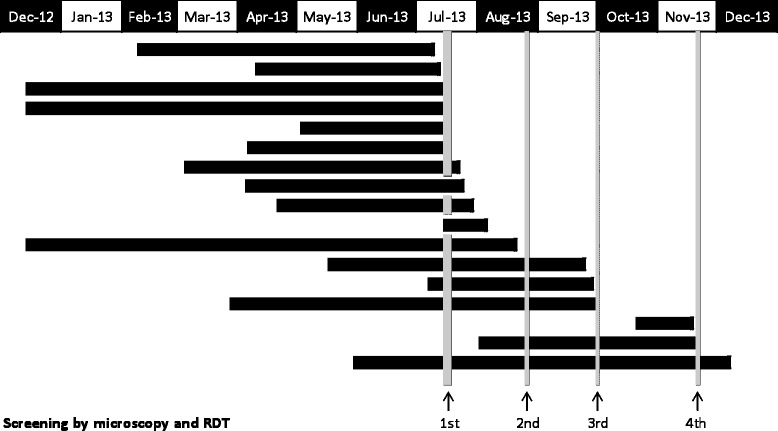
Duration of stay in Sri Lanka of each malaria case (bar) from their date of arrival to Sri Lanka until the date of detection/diagnosis by either passive case detection or active case detection during screening programmes (dates of screening done by the AMC HQ are indicated by gray vertical lines).

Between July and December 2013 a further seven individuals, who had been screened at least once during the ACD programme and tested negative for malaria, presented with fever to the same DGH and were diagnosed with *P. viv*ax malaria. Additionally, three individuals who were not available for screening by any of the four ACD programmes were also diagnosed with malaria after presenting with fever; one patient gave a history of being treated for dengue as an inpatient in a government hospital and being discharged without a blood smear examination for malaria parasites, another was reported from a teaching hospital in the North Central Province (which is a previously high malaria endemic area) and one individual had arrived in the country and was diagnosed between screening programmes. By the end of December 2013, there was a total of 17 Pakistanis, some of who had been living for as long as 9 months in Sri Lanka prior to being diagnosed with malaria. Six of them gave a confirmed past history of malaria prior to arriving in Sri Lanka. All the others reported episodes of fever prior to arrival and had been treated in Pakistan, although neither the exact dates nor the details of medication could be elicited with any accuracy. A summary of the number of patients diagnosed with malaria is given in Table 1. Figure 1 indicates the date of arrival of the asylum seekers to Sri Lanka, the time periods at which the ACD programmes were carried out and the date of diagnosis of malaria infections in individuals who tested positive for parasites. In 16 of the 17 malaria-infected Pakistanis, *P. vivax* gametocytes were seen on Giemsa-stained thick and thin blood smears. In addition to these 17 cases reported in 2013 which are described above, a further six vivax malaria cases were reported sporadically from this group of Pakistani asylum seekers during 2014. All individuals diagnosed with malaria were treated as in-ward patients with the nationally recommended weight-appropriate doses of chloroquine (25 mg/kg bodyweight) for three days followed by a 14-day course of primaquine (0.25 mg/kg/day) (excluding the pregnant mother for the latter) under close medical supervision for symptoms and signs of acute haemolytic anaemia. They were followed up according to the national guidelines on days 3, 7, 14, 21, and 28.

**Table 1:** **Malaria infections reported amongst the Pakistani asylum-seekers between July and December 2013**

**Patient no.**	**Age**	**Sex**	**Malaria species**	**Stage/s**	**Summary of clinical history**
1	8 yrs	F	Pv	Trophozoites (T)/Gametocytes (G)	Developed fever on the 3^rd^ of July 2013, admitted to a District General Hospital (DGH) in the Western Province on the 8^th^ of July and blood smear tested positive for malaria parasites on the 9^th^ of July.
2	11 yrs	F	Pv	T/G	Fever for three days prior to admission to the DGH on the 12^th^ of July and diagnosed with malaria on the same day.
**Active case detection (ACD) for malaria carried out among the refugee population by microscopy and RDT by Public Health Laboratory Technicians (PHLTs) of the Anti Malaria Campaign from the 15** ^**th**^ **– 18** ^**th**^ **July 2013.**					
3*	42 yrs	M	Pv	T/G	Identified by ACD. Asymptomatic at the time of diagnosis. History of fever two days ago. Sought treatment from a General Practitioner but no blood smear done for diagnosis of malaria. Developed fever with chills and rigors immediately after admission to hospital.
4*	38 yrs	F	Pv	T/G	Identified by ACD. Asymptomatic at the time of diagnosis. History of Irregular fever over past 16 days. Sought treatment but no blood smear done for malaria diagnosis. Wife of case number 3.
5*	31 yrs	F	Pv	T/G	Identified by ACD. Asymptomatic at the time of diagnosis. 32 weeks pregnant. Flu like illness with chills over past one month. Received treatment for a urinary tract infection but no blood smear done for malaria diagnosis.
6*	7 yrs	M	Pv	T/G	Identified by ACD. Asymptomatic at the time of diagnosis. Irregular occurrence of fever over past 14 days. Sought treatment but no blood smear for malaria diagnosis.
7**	35 yrs	M	Pv	T/G	Screened by ACD and found to be negative. Presented with fever on the 22^nd^ of July to the same DGH and was found to be positive for malaria.
8	22 yrs	M	Pv	T/G	Reported from the Teaching Hospital Anuradhapura in the North Central Province, a former high malaria endemic area. Originated from the same neighbourhood but working in this district for the past one month (was not present during ACD). Diagnosed with malaria on the 24^th^ of July.
9**	9 yrs	M	Pv	T/G	Screened by ACD and found to be negative for malaria. Fever commenced one week after ACD on the 25^th^ of July. Diagnosed on the 29^th^ of July at the DGH.
10	25 yrs	F	Pv	T/G	Not screened by ACD by the PHLTs. Admitted to the same DGH 3 weeks later on the 5^th^ of August with fever and diagnosed with malaria. Had been admitted to same hospital 10 days prior to this, been treated for dengue and discharged without having a blood smear examined for malaria parasites.
11**	2 yrs	M	Pv	T/G	The child of case number 3 and 4 who was found to be negative for malaria parasites by ACD. Admitted with fever on 18^th^ of August to the DGH and diagnosed with malaria on the 20^th^ of August.
**Active case detection (ACD) for malaria carried out among the refugee population by microscopy and RDT by Public Health Laboratory Technicians (PHLTs) of the Anti Malaria Campaign on the 26th of August 2013.**					
12**	29 yrs	M	Pv	T/G	Screened by ACD and found to be negative for malaria. Presented with fever to the DGH and diagnosed with malaria on the 24^th^ of September 2013.
13**	28 yrs	M	Pv	T/G	Screened by ACD and found to be negative for malaria by microscopy and RDT. Presented with fever to the DGH and diagnosed with malaria on the 28^th^ of September.
14**	23 yrs	M	Pv	T	Screened during ACD done in July and found to be negative. Developed fever on the 28^th^ of September and diagnosed with malaria on the 1^st^ of October at the DGH.
**Active case detection (ACD) for malaria carried out among the refugee population by microscopy and RDT by Public Health Laboratory Technicians (PHLTs) of the Anti Malaria Campaign on the 1** ^**st**^ **of October 2013.**					
15	24 yrs	F	Pv	T/G	Arrived in Sri Lanka after last screening. History of irregular fever since the 2^nd^ of November and diagnosed with malaria on the 18^th^ of November.
16*	5 1/2 yrs	F	Pv	T/G	Identified by ACD. Asymptomatic at the time of diagnosis. History of irregular fever since the 1st of November and diagnosed with malaria on the 21st of November.
**Active case detection (ACD) for malaria carried out among the refugee population by microscopy and RDT by Public Health Laboratory Technicians (PHLTs) of the Anti Malaria Campaign on the 21** ^**st**^ **of November 2013**					
17**	22 yrs	M	Pv	G	Screened during ACD and found to be negative. History of irregular fever since the 4^th^ of December and diagnosed with malaria on the 7^th^ of December.

*Diagnosed by active case detection by contact tracing.

**Negative by active case detection but presented with fever on a later date and diagnosed with malaria.

### Cluster of *Plasmodium falciparum* infections amongst Sri Lankan fisherman returning from Sierra Leone

A group of 14 Sri Lankan fishermen originating from traditionally malaria non-endemic areas of Sri Lanka, who were employed in fishing trawlers in Sierra Leone for four months, returned to the country on the 14^th^ of August 2013. Following the diagnosis of *P. falciparum* malaria on 18^th^ of August in one of the fishermen, tracing of contacts and screening by ACD was commenced by the AMC HQs. Another eight *P. falciparum* malaria positive cases were diagnosed by microscopy and/or RDT amongst these returnees. Of these nine patients, three developed severe malaria (Table 2).

**Table 2:** **Summary of patients who arrived from Sierra Leone, Africa and tested positive for malaria**

**Patient no.**	**Age**	**Sex**	**Malaria species**	**Stage/s**	**Summary of clinical history**
1	55 yrs	M	Pf	Ring stage (R)	Fever for three days since the 15^th^ of August. Admitted to a tertiary care Teaching hospital in the Western Province (traditionally malaria non-endemic area) and diagnosed with malaria by microscopy and RDT on the 18^th^ of August
2*	47 yrs	M	Pf	R	Identified by ACD. On tracing patient had been admitted with fever, chills and rigors to a hospital in a previous malaria endemic area on the 12^th^ of August. Malaria blood smear had not been done in-spite of giving a history of returning from a malaria endemic country until informed by AMC HQs. Found to be positive on the 19^th^ of August. Following first dose of ACT patient developed severe malaria with haematuria and drowsiness. Treated with IV quinine in the intensive care unit and made full recovery without sequale.
3*	49 yrs	M	Pf	RDT + Microscopy negative	Identified by ACD. Asymptomatic at the time of diagnosis on the 19^th^ of August. Patient refused to get admitted following diagnosis and was treated by DOTS at home.
4*	47 yrs	M	Pf	R	Identified by ACD. Asymptomatic at the time of diagnosis on the 21^st^ of August. Admitted to hospital and treated by DOTS.
5*	55 yrs	M	Pf	R/G	Identified by ACD. On tracing back had been admitted with fever on the 14^th^ of August to the DGH where the Pakistanis were treated. Malaria blood smear had not been done until informed by AMC HQs. Positive for malaria and treated with ACT on the 21^st^ of August. However patient developed severe malaria with haematuria. Treated with IV quinine.
6*	36 yrs	M	Pf	R	Identified by ACD. Asymptomatic at the time of diagnosis on the 21^st^ of August. Admitted to hospital and treated with DOTS.
7*	42 yrs	M	Pf	R	Identified by ACD. Asymptomatic at the time of diagnosis on the 21^st^ of August. Admitted to hospital but in spite of DOTS developed severe malaria with drowsiness. Treated with IV quinine.
8*	47 yrs	M	Pf	R	Identified by ACD. Asymptomatic at the time of diagnosis on the 22^nd^ of August. Developed fever following admission. Treated with DOTS.
9*	43	M	Pf	R	Identified by ACD. Febrile at the time of diagnosis on the 22nd of August. Treated with DOTS.
**2** ^**nd**^ **batch of fishermen from Sierra Leone arrived in Sri Lanka on the 24** ^**th**^ **of December 2013**					
10	42 yrs	M	Pf	R	Presented to the AMC HQs on the 27^th^ of December with a history of being diagnosed with falciparum malaria prior to leaving Sierra Leone. Was on anti-malarial treatment (different regime of ACT - not familiar in Sri Lanka). RDT positive/microscopy negative for falciparum malaria when tested at AMC. Diagnosis confirmed by PCR. Treated with ACT and Primaquine.
11	40 yrs	M	Pf	R	Had been screened for malaria by microscopy and RDT on the 27^th^ of December at AMC HQs and tested negative. Following admission to NHSL^α^ with fever on the 30^th^ of December was diagnosed with malaria.
12*	48 yrs	M	Pf	R	Had been screened for malaria by microscopy and RDT on the 27^th^ of December at AMC HQs and tested negative. At the time of tracing patient was admitted to a Teaching Hospital in the Western Province but had not been tested for malaria. Diagnosed on the 1^st^ of January 2014.
13*	38 Yrs	M	Pf	R	Had been screened for malaria by microscopy and RDT on the 27^th^ of August at AMC HQs and tested negative. At the time of tracing patient was admitted to a different ward in the same Teaching hospital as patient no. 12 but had not been tested for malaria. Diagnosed with malaria on the 1^st^ of January 2014.

*Diagnosed by active case detection by contact tracing.

^α^NHSL: National Hospital of Sri Lanka.

Another of 11 fishermen, returned to Sri Lanka on the 24^th^ of December 2013, after a stay of 19 days in Sierra Leone. One individual had developed malaria and started treatment with Artemisinin Combination Therapy (ACT) prior to leaving Sierra Leone. He presented to the AMC HQs on the 27^th^ of December 2013 and was treated for malaria as he was positive for malaria by RDT (negative by microscopy but diagnosis confirmed by PCR). The five colleagues who accompanied him to the AMC HQs were negative for malaria parasites (by RDT and microscopy) on that day, but three of them developed the disease at a later stage (which was confirmed by microscopy), giving a total of 13 cases of falciparum malaria being reported from this group. All of them had been given chemoprophylaxis for malaria prior to departure but compliance had been poor.

**Figure 2:**
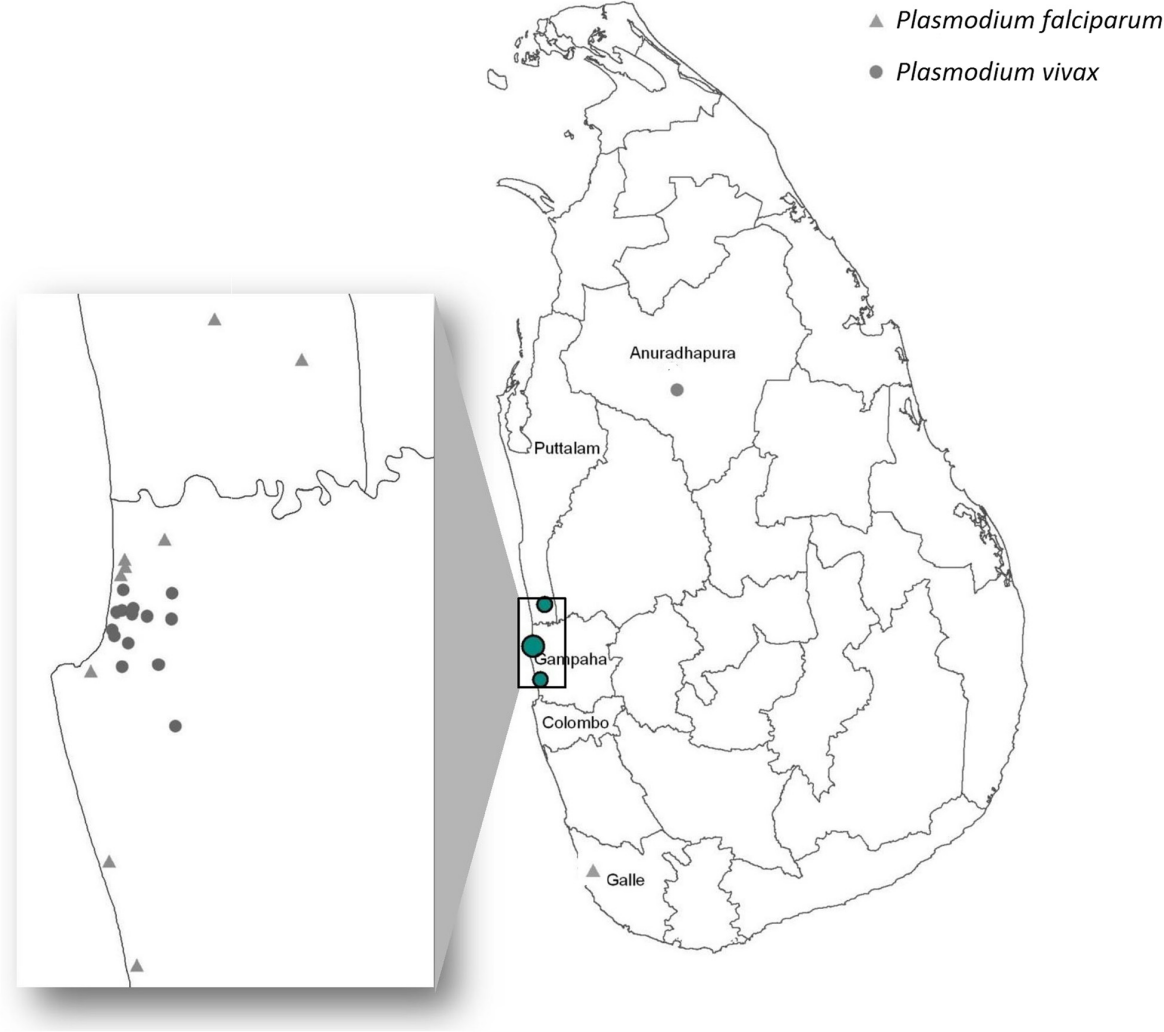
Location of residence of Pakistani asylum seekers and fishermen from Sierra Leone who tested positive for malaria parasites during the period July 2013- January 2014.

A summary of the history of illness, species and stages of parasites detected is given in Table 2. Figure 2 indicates the site of residence of the Pakistani asylum seekers and the fishermen from Sierra Leone.

Both clusters of infected persons were residing in typically semi-urban areas (Figure 2). In response to these clusters of cases, five entomological surveys (adult and larval mosquito surveys) were carried out in all potential mosquito breeding places in the neighbourhood. *Anopheles culicifacies,* the primary vector for transmission of malaria in Sri Lanka was not found by adult and larval sampling techniques but small numbers of larval and adult stages of *Anopheles subpictus* and *Anopheles varuna* which are potential malaria vectors were collected with no infective stages reported after dissections.

The evidence available following comprehensive epidemiological, entomological, and parasitological investigations support the classification of these cases as imported as these individuals arrived from malaria endemic countries [5]. Moreover, these cases are unlikely to be due to indigenous transmission due to: (i) the geographical area where both populations have been residing is traditionally a non-malarial area, (ii) the entomological investigations done following the reporting of these cases indicate that primary vectors responsible for malaria transmission in Sri Lanka were not present, (iii) no infective stages were found in the secondary vectors that were detected, (iv) repeated parasitological screening carried out amongst the surrounding local community did not detect any malaria positive cases, (e) curative health care institutions in the area of drainage (both private and government including general practitioners) has not encountered any indigenous malaria cases for approximately two years.

### Malaria infections reported among other high-risk groups

In addition to the malaria cases reported in the above two groups, there were other clusters of cases that were reported among high-risk populations in 2013/2014. This included four *P. falciparum* cases reported from Sri Lankan army personnel returning after serving as peace-keeping forces in Haiti and South Sudan, three malaria cases reported among irregular migrants from Myanmar being kept at detention camps in the Western Province and 15 malaria positives being identified by active and passive case detection in groups of foreign skilled and unskilled labourers, working in several parts of the country. In addition to the clusters mentioned above there were sporadic cases reported from various parts of the country, including in Sri Lankan nationals engaged in business in African countries and India. Over this period 2013–2014, a total of 96 Sri Lankan nationals and 48 foreign nationals were diagnosed with imported malaria.

## Discussion

With zero indigenous disease burden, prompt case detection by health care providers is a major challenge, both amongst individuals returning from malaria endemic countries and the local population as malaria is low on the differential diagnosis of patients presenting with fever. This is further complicated by the dengue epidemic looming in the country which is a priority in the protocol of differential diagnosis when a patient presents with fever and/or thrombocytopenia [6]. This has resulted in unacceptable delays, sometimes exceeding 30 days to diagnose malaria since the onset of fever [4]. Extended delays in diagnosis will not only increase the disease transmission potential but would also increase the risk of the person developing severe disease with an associated risk of mortality. Fifteen persons amongst the 144 imported malaria cases reported over this two year period developed severe disease as a result of delayed diagnosis all of who recovered without residual disability.

With a view of addressing this issue, the AMC has developed and distributed throughout the country new treatment guidelines and protocols emphasizing the identification and management aspects to prevent progression to severe disease [7]. To enhance malaria surveillance activities stocks of RDTs and diagnostics have been supplied to areas where a high number of imported malaria cases are being reported. In order to ensure that the anti-malarial medicines are not freely available for inadvertent use by the health care providers, these medicines are supplied through the AMC network to reach any part of the country well within two hours of need. The AMC continues awareness programmes for health care providers, military personnel, travel agents and hoteliers island-wide, and do special in-service training programmes for laboratory technicians of the government and private hospitals.

There were individuals who tested negative for malaria during previous screening programmes conducted by the AMC HQs (this includes 7/17 Pakistanis and 3/13 returnees from Sierra Leone) but developed clinical disease subsequently. The reasons for not detecting malaria parasites at the time of active surveillance, in spite of these individuals being screened by competent Public Health Laboratory Technicians (PHLTs) of the Anti Malaria campaign could be that: (i) in the case of the Pakistanis the presenting *P. vivax* infections may have been relapses due to the activation of dormant liver stages, (ii) the fishermen who presented with *P. falciparum* infections may have been in the pre- patent period when they were initially screened, (iii) partial treatment with anti-malarials in Pakistan or irregular use of chemoprophylaxis by the fishermen may have led to sub-microscopic levels of parasites which could not be detected by microscopy at the time of the initial screening, but resulted in higher parasite densities and a patent infection later.

A compelling question that needs to be answered with each malaria infection being reported in the country is whether these cases could be due to indigenous transmission of malaria. For a country which has achieved elimination and entering into prevention of reintroduction phase, this question would be extremely critical as the implications of determining whether it is an imported or indigenous case would have completely different approaches with regard to further actions and management. An imported malaria infection is defined as a case of malaria that has been acquired in another country and diagnosed in Sri Lanka by blood smear examination and/or RDT [3]. As these clusters were classified as imported cases, the requirement would be to ensure that they no longer contribute towards the development of an active focus. Although these clusters were reported from traditionally non-malaria endemic areas there is a potential risk of transmission due to the presence of the secondary vector. In contrast, had they been labelled as indigenous cases the country’s claim to a malaria free status since November 2012 will be challenged. The AMC took additional active measures at transmission control in a focal manner.

The threat posed by imported malaria to reintroducing the disease to Sri Lanka is quite conceivable. The mechanisms available to the host country to protect its population against imported malaria include routine screening of high-risk communities and presumptive treatment. Voluntary screening and treatment mechanisms for malaria are operational at ports of entry, including Bandaranaike International Airport. Large groups of individuals arriving from malaria endemic countries, including security personnel returning from United Nations peace keeping missions are screened for malaria when ever their arrival is notified to the AMC. This strong collaboration between the AMC and the Sri Lanka army makes it possible to diagnose early, imported malaria infections in this high-risk group. However, active screening of immigrants, such as the Pakistani asylum seekers and foreign workers who enter on a tourist visa and thereafter settle down in the country, is hardly feasible unless collaborating institutions such as the UNHCR or International Organization for Migration (IOM) informs the AMC of new arrivals. The UNHCR provides advice, assistance and international protection to persons that it recognizes as refugees but a pre-requisite for such services would be initial registration with the Organization. Therefore details of irregular migrants who are yet to register with them will not be available. A similar situation also exists with the IOM indicating that collaboration with these organizations will not make it possible to reach all members of these high-risk groups to implement customary malaria control interventions due to difficulties in identifying them. Further, individuals such as this may go into hiding if they are not documented as residents and cannot be found for follow-up. New entrants may not settle down in the same location as their precedents making it a daunting task to locate and access them. Enhanced surveillance by repeated screening using microscopy and/or RDT through advocacy among the community and religious leaders of these asylum seekers played a major role in early identification of malaria cases. Sensitivity and empathy towards these groups were important in obtaining their cooperation to make active surveillance a success. These individuals do not voluntarily come into the screening procedure, especially when they consider themselves as healthy. Even individuals who have undergone a screening test once and tested negative for malaria, develop a false sense of security that persuading them for another repeat test later, or even when they develop fever, is a challenging task.

Presumptive treatment of all members of migrant groups who have been identified as having a high likelihood of being infected with malaria from their country of origin would have been considered as an option. However, considering the level of compliance needed to achieve the desired outcome, the cost, feasibility, risk of developing resistance to ACT and potential adverse reactions following primaquine administration, the AMC resorted to intensified surveillance among these high-risk groups. The risks of identifying them as a “malaria risk group” may include creating ethnic disharmony leading to cultural sensitivities and even jeopardizing diplomatic relations between countries. Intensified surveillance has been deemed necessary to ensure that every infection is detected to prevent the risk of re-establishment through imported malaria [8].

The Anti Malaria Campaign continues to rapidly respond to outbreaks with respect to surveillance, clinical management, laboratory diagnosis, entomological surveillance, vector control and communication in order to prevent transmission of the disease. Sri Lanka has eliminated malaria and support has been pledged at the highest level of government to prevent its reintroduction.

## Conclusions

With no indigenous malaria cases being reported in Sri Lanka since November 2012, the country is at a critical juncture in its efforts to prevent the reintroduction of malaria. With the aim of seeking certification as malaria-free from the World Health Organization by 2016, the main thrust of Sri Lanka’s National Strategic Plan for 2014–2018 for malaria elimination and prevention of reintroduction is planning and implementing a comprehensive programme on intensified surveillance with strong emphasis on early detection and treatment of imported malaria to ensure there is no further spread.

As illustrated by this report, imported malaria is the biggest threat to sustaining a malaria free country, and poses a potential risk of reintroduction of the disease to Sri Lanka. Receptivity to malaria in previously malaria endemic areas of the country is high due to the prevalence of the vector mosquito and therefore, persons with imported malaria travelling to these areas could result in a focus of local transmission particularly because the population of Sri Lanka would have lost immunity to malaria over the past several years. Based on the information presented in this paper, the AMC has profiled high-risk groups for targeting malaria surveillance activities such as military personnel returning from peace keeping missions, through a collaboration with the armed forces. Yet the identification of other high-risk groups may not be possible until a member of the group presents and is diagnosed with malaria, which provides a lead as was seen in the two clusters reported here. This report also illustrates that malaria diagnoses is being delayed in spite of a very good coverage of diagnostic services (microscopy and RDT) throughout the country, even when patients present with fever and a history of travel to a malaria endemic country, this being largely due to malaria being low in the differential diagnosis of a patient presenting with fever. The AMC continues to take multiple appropriate measures by way of screening programmes through collaborations with UN agencies, port authorities, religious leaders associated with asylum seekers, and conducting awareness and training programmes within the health sector. To prevent the reintroduction of malaria into Sri Lanka, these measures are combined with a highly rigorous response arm of the Campaign when a case is detected, as is described in this report.

## Abbreviations

ACD: Active case detection
ACT: Artemisinin-based combination therapy
AMC: Anti Malaria Campaign
AMC HQs: Anti malaria campaign headquarters
DGH: District General Hospital
NHSL: National Hospital of Sri Lanka
PCR: Polymerase chain reaction
PHLT: Public Health Laboratory Technicians
RDT: Rapid diagnostic test
